# Field and Thermal Emission Limited Charge Injection in Au–C60–Graphene van der Waals Vertical Heterostructures for Organic Electronics

**DOI:** 10.1021/acsanm.3c01090

**Published:** 2023-05-22

**Authors:** Jacopo Oswald, Davide Beretta, Michael Stiefel, Roman Furrer, Sebastian Lohde, Dominique Vuillaume, Michel Calame

**Affiliations:** † Transport at Nanoscale Interfaces Laboratory, Empa - Swiss Federal Laboratories for Materials Science and Technology, Überlandstrasse 129, CH-8600 Dübendorf, Switzerland; ‡ Swiss Nanoscience Institute, University of Basel, Klingelbergstrasse 82, CH-4056 Basel, Switzerland; § Department of Physics, University of Basel, Klingelbergstrasse 82, CH-4056 Basel, Switzerland; ∥ Institute of Electronic, Microelectronic and Nanotechnology (IEMN), Centre National de la Recherche Scientifique, Villeneuve d’Ascq 59652, France

**Keywords:** organic, semiconductor, graphene, interface, transport, vertical, van der Waals, C60

## Abstract

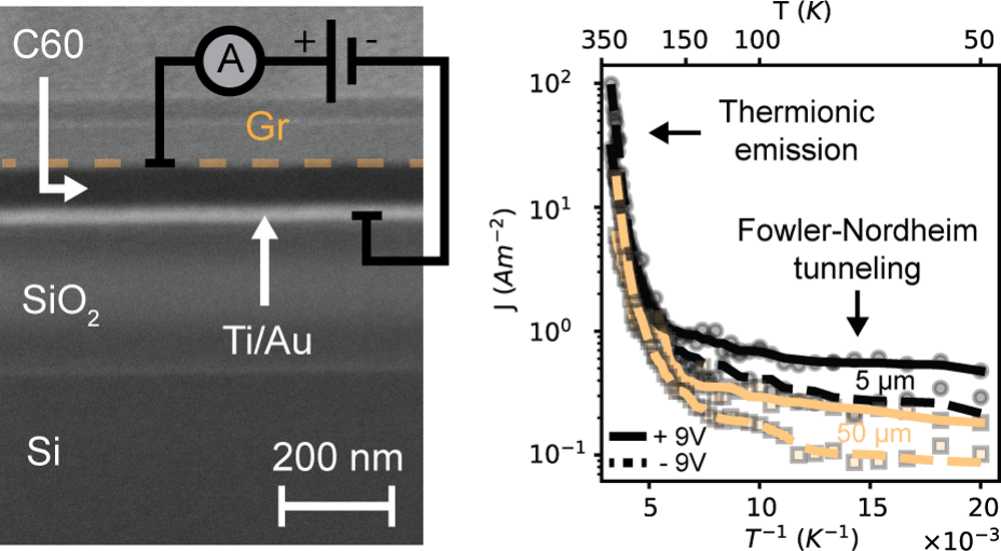

Among the family of 2D materials, graphene is the ideal candidate as top or interlayer electrode for hybrid van der Waals heterostructures made of organic thin films and 2D materials due to its high conductivity and mobility and its inherent ability of forming neat interfaces without diffusing in the adjacent organic layer. Understanding the charge injection mechanism at graphene/organic semiconductor interfaces is therefore crucial to develop organic electronic devices. In particular, Gr/C60 interfaces are promising building blocks for future n-type vertical organic transistors exploiting graphene as tunneling base electrode in a two back-to-back Gr/C60 Schottky diode configuration. This work delves into the charge transport mechanism across Au/C60/Gr vertical heterostructures fabricated on Si/SiO_2_ using a combination of techniques commonly used in the semiconductor industry, where a resist-free CVD graphene layer functions as a top electrode. Temperature-dependent electrical measurements show that the transport mechanism is injection limited and occurs via Fowler–Nordheim tunneling at low temperature, while it is dominated by a nonideal thermionic emission at room and high temperatures, with energy barriers at room temperature of ca. 0.58 and 0.65 eV at the Gr/C60 and Au/C60 interfaces, respectively. Impedance spectroscopy confirms that the organic semiconductor is depleted, and the energy band diagram results in two electron blocking interfaces. The resulting rectifying nature of the Gr/C60 interface could be exploited in organic hot electron transistors and vertical organic permeable-base transistors.

## Introduction

Graphene is the ideal candidate as top or interlayer electrode for hybrid van der Waals heterostructures made of organic thin films and 2D materials due to its high conductivity and mobility and its inherent ability of forming neat interfaces without diffusing in the adjacent organic layer, in contrast to commonly used metal electrodes.^1^ Graphene–organic hybrid devices have been widely explored^2^ for their potential use in various applications, including organic light-emitting diodes,^3,4^ organic photovoltaics,^5,6^ and vertical organic transistors.^7−12^ Nevertheless, new techniques and large-area fabrication methods still need to be developed for effectively incorporating graphene as a top and/or interlayer electrode in hybrid organic devices. In fact, graphene is typically implemented as a gateable bottom electrode into barristors.^7−12^ However, more complex multilayer architectures could exploit the unique properties of graphene, i.e., optical transparency,^13^ flexibility,^14^ and the ability to conform to three-dimensional and/or rough surfaces.^15^ For instance, graphene could be implemented as the base electrode in organic hot electron transistors, where ultrathin organic layers are required, or replace the metallic base electrode in vertical organic permeable-base transistors.^16,17^ This would enable flexible vertical organic transistors operating at high frequencies^18^ that are suitable for applications such as wearable electronics with communication functions, or RF tags. Flexible vertical transistors could also be vertically integrated in OLEDs,^16^ enabling high-resolution organic displays with enhanced contrast. Within this framework, understanding the charge transport mechanisms occurring at the interface between graphene and organic semiconductors (OSC) is crucial for the development of new graphene-based optoelectronic devices.^2,19,20^

The fabrication techniques presented in this work are similar to what previously reported for vertical VdW heterostructures made of the p-type polymer P3HT and CVD grown graphene.^21^ In this case, however, the graphene sheet is transferred on top of a thermally evaporated thin film of small molecules, i.e., the n-type organic semiconductor C60, and subsequently patterned into the top electrode. The fabrication processes combines techniques commonly used in the semiconductor industry, and therefore it can be upscaled to wafer level. The charge injection mechanisms at the interfaces, i.e., Gr/C60 and Au/C60, are investigated by temperature-dependent *I*–*V* measurements and impedance spectroscopy. The current–voltage characteristic is modeled by the double Schottky barrier (DSB) model at room and high temperature (above 300 K) and by Fowler–Nordheim (FN) tunneling at low temperature (below 100 K). The energy barrier height at the Gr/C60 and Au/C60 interfaces is extracted from the DSB and from the FN tunneling models, while the static dielectric constant of C60 is determined by impedance spectroscopy. The study concludes with the energy band diagram of the heterostructure explaining the charge injection at the two interfaces.

## Results and Discussion

**Figure 1:**
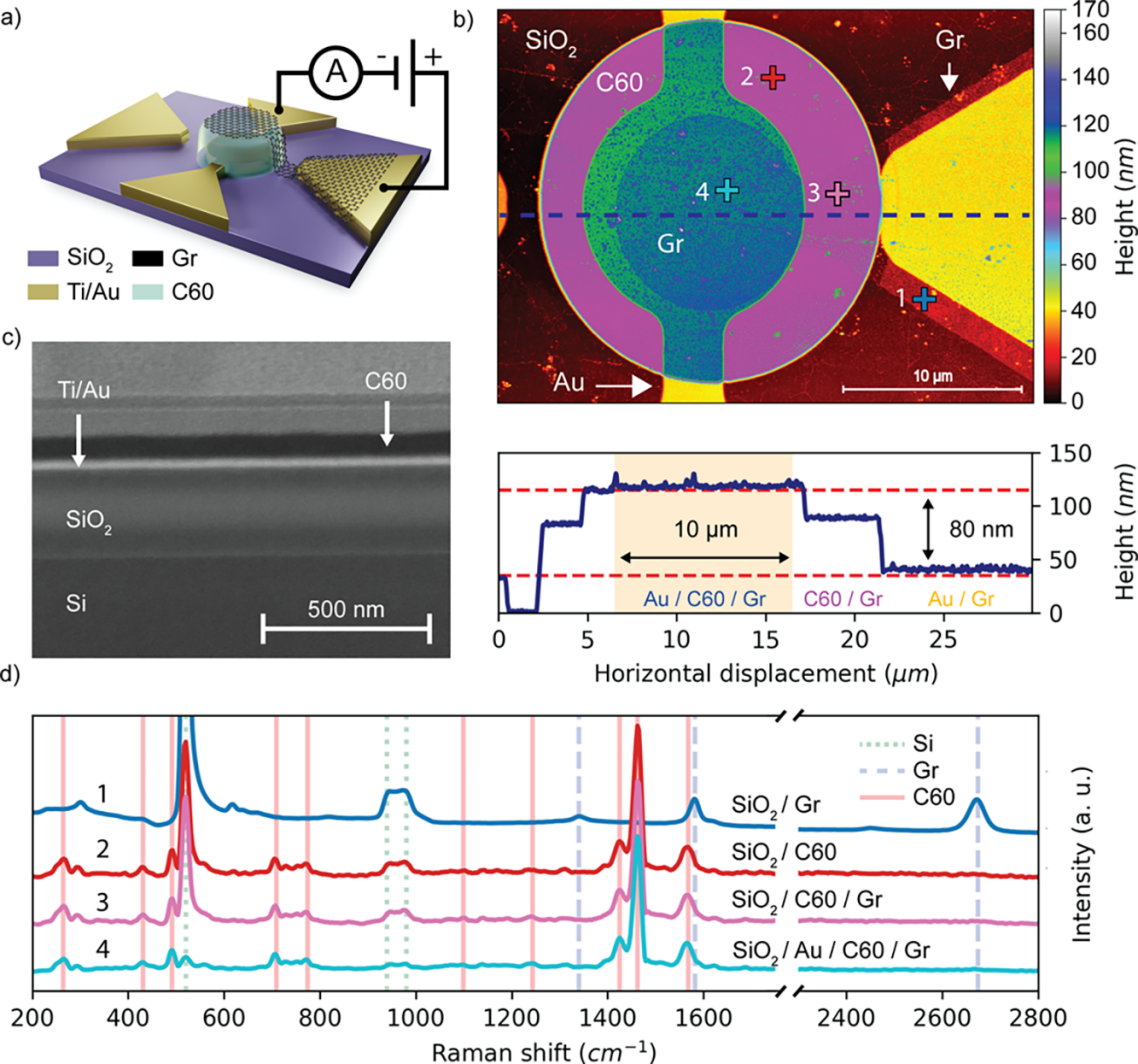
(a) 3D schematic of the Au/C60/Gr vertical heterostructure (not to scale). Adapted from ref (21). (b) AFM height image and profile of a representative 10 μm device. (c) Cross-section SEM image of the Au/C60/Gr heterostructure cut at the center of the graphene top electrode, parallel to the AFM profile shown in (b). (d) Raman spectra taken at different locations of the device, marked by colored crosses in (b). The vertical lines with different colors refer to the characteristic Raman peaks of Si, Gr, and C60.

This study was performed on one chip that includes two distinct groups of devices: (i) 119 Au/C60/Gr *Vertical Stacks*, with diameters spanning from 5 to 50 μm and C60 film thickness of 80 nm, and (ii) 34 *Graphene Bridges* to characterize the in-plane resistance of graphene after transfer on C60. Figure 1a shows the 3D schematic of the Au/C60/Gr *Vertical Stack*, fabricated following the protocol reported in the Experimental Methods and in the Supporting Information, together with the electrical scheme implemented for the measurements, while the schematic of the *Graphene Bridge* and the corresponding electrical scheme are shown in the Supporting Information (Figure S1). Figure 1b shows the AFM height image and height profile of a representative *Vertical Stack* having a diameter of 10 μm. From the AFM height image one can extract the actual diameter of the graphene electrode (i.e., 10 μm), while the height profile shows the thickness of the bottom electrodes (35 nm) and of C60 (80 nm). Refer to the Supporting Information for the AFM height image of a representative *Graphene Bridge* device (Figure S1). Figure 1c shows a cross section of the Au/C60/Gr stack, where the four visible layers are Si (525 μm), SiO_2_ (335 nm), Ti/Au (5 nm/30 nm), and C60 (80 nm). The resolution of the instrument does not allow to observe the graphene electrode on top of the stack. Figure 1d shows the Raman spectra taken at different locations of the device, marked by colored crosses in Figure 1b. The Raman spectrum of CVD graphene (SiO_2_/Gr) shows the characteristic G (1582 cm^–1^) and 2D (2674 cm^–1^) peaks, as well as the D (1340 cm^–1^) peak, with a weak amplitude, possibly resulting from fabrication-induced defects. The C60 film (SiO_2_/C60), the C60 film covered by graphene (SiO_2_/C60/Gr), and the full stack (SiO_2_/Au/C60/Gr) Raman spectra are virtually identical and display the characteristic Raman-active vibrations of C60,^22^ i.e., H_g_ (1) at 264 cm^–1^, H_g_ (2) at 431 cm^–1^, A_g_ (1) at 492 cm^–1^, H_g_ (3) at 709 cm^–1^, H_g_ (4) at 774 cm^–1^, H_g_ (5) at 1099 cm^–1^, H_g_ (6) at 1244 cm^–1^, A_g_ (2) at 1463 cm^–1^, and H_g_ (8) at 1568 cm^–1^. The typical Raman scattering of Si,^23^ i.e., the first-order optical mode (520 cm^–1^) and second-order scattering band (940–980 cm^–1^), due to the Si substrate, is observed in all spectra. The results of the AFM, SEM, and Raman analysis show that the methods used to fabricate the vertical VdW devices are compatible with graphene and C60 and do not degrade these materials.

**Figure 2:**
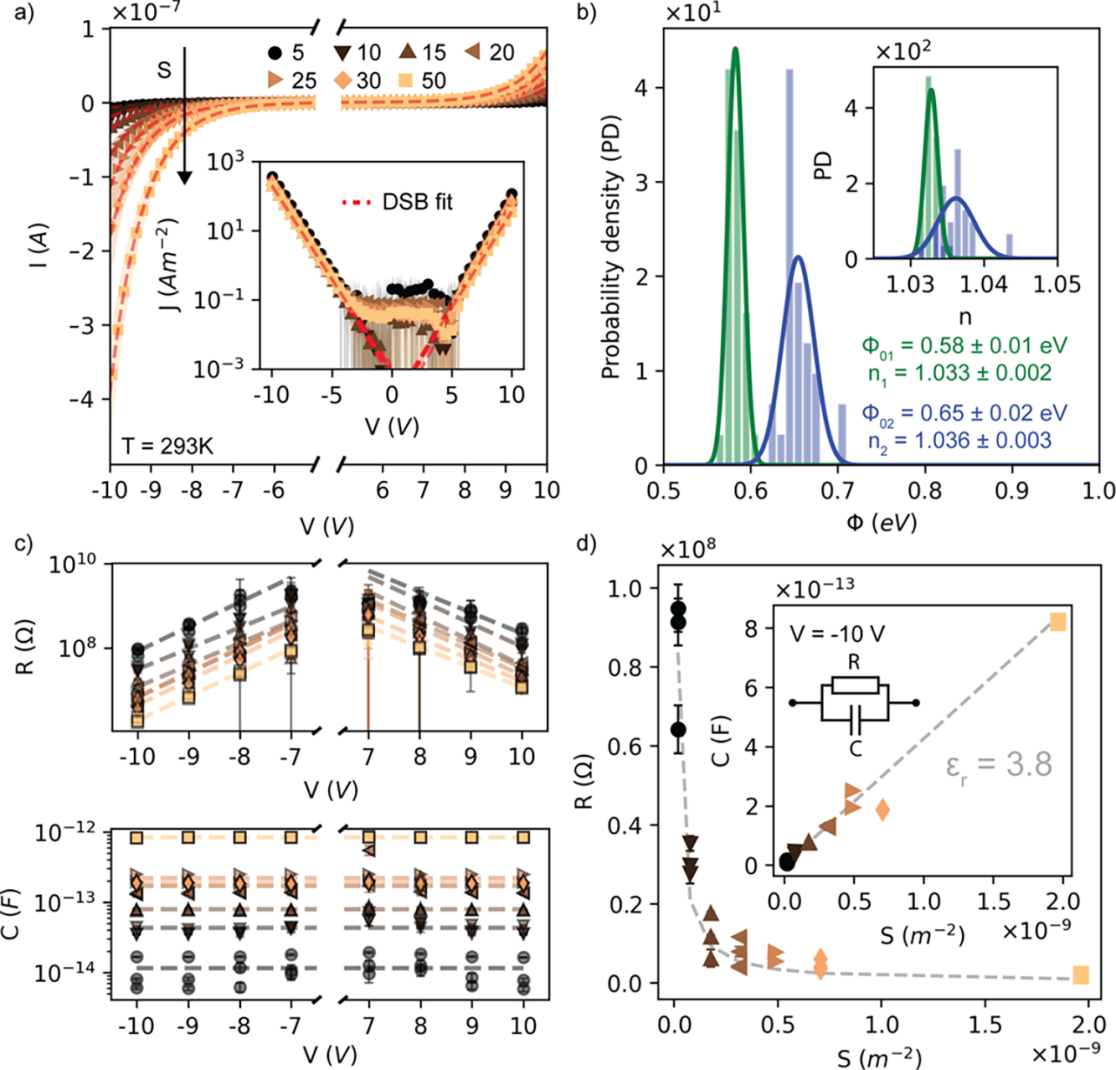
Electrical measurements under vacuum (∼10^–6^ mbar) at room temperature (293 K). (a) Average of the current–voltage characteristics measured on five devices for each diameter: 5, 10, 15, 20, 25, 30, and 50 μm. The shaded area represents the standard deviation. The dashed red line is the average of the DSB fits on five devices per area. The figure does not display the *I*–*V* data in the range from −5 to +5 V, where the current is below the sensitivity of the instrument. The inset shows the *J*–*V*s on the whole measurement range. (b) Distribution of Φ_01,02_ and *n*_1,2_ extracted from the DSB on 35 device (five devices for each diameter), associated with the Gr/C60 and Au/C60 interfaces, respectively. (c) Resistance *R* and capacitance *C* vs applied bias, extracted from the fit of the nonideal capacitor model. The dashed lines are the linear fit of *R* and *C* versus bias. (d) Scaling of *R* and *C* with device area. The inset shows the capacitance *C* vs the device area *S*. The gray dashed lines represent the capacitance *C* = ϵ_0_ϵ_*r*_*S*/*t*, where ϵ_*r*_*=* 3.8 is the deduced static dielectric constant of C60, and the resistance *R*, which scales as 1/*S*.

The effects of the environment and of the device area on the charge transport are investigated by comparing the *I*–*V* characteristics measured in air and a vacuum and by studying the scaling relationship between the system resistance and capacitance and the device area, where the device resistance and capacitance are obtained from impedance measurements under vacuum. Oxygen and moisture are known to affect the electrical properties of C60 and graphene.^24−29^ In particular, oxygen and moisture typically act as electron trap centers for C60, reducing the electrical conductivity of the film,^27−29^ while they typically p-dope graphene on SiO_2_,^24−26^ as shown in the Supporting Information for the *Graphene Bridges* devices (Figure S2 and Table S1) and as demonstrated on graphene field effect transistors (Figure S3). In order to desorb oxygen and water from C60 and from the graphene surface, and therefore ensure the consistency and reproducibility of measurements done in different systems, all measurements are done in a vacuum after annealing (Figure S4). Figure 2a shows the average *I*–*V* traces of five devices per area measured under vacuum (∼10^–6^ mbar) at room temperature (293 K) after annealing for 12 h at 110 °C. For the purpose of clarity, the figure does not display the *I*–*V* data in the range from −5 to +5 V, where the current is below the sensitivity of the instrument. The inset of Figure 2a shows that the *J*–*V* traces of all devices overlap and, thus, that the transport characteristic is not affected by the device area. The small variability between *J*–*V*s is ascribed to the device inhomogeneities due to the fabrication. After annealing, the *J*–*V* characteristics become very similar, while a larger variability is observed for the *J*–*V* traces taken in air and shown in Figure S5. Interestingly, the stacks become less conductive in a vacuum after annealing (Figure S4), against what one would expect if the charge transport were bulk limited by C60 due to oxygen and moisture desorption. This observation suggests that the transport is not bulk-limited but injection-limited and that the oxygen and moisture have an impact on the energetics of the interfaces. All *J*–*V*s display the same nonlinear and asymmetric S-shaped characteristic: the current initially increases slowly with increasing voltage and then rapidly increases at higher voltages; the same occurs for negative voltages, but the magnitude of the current is higher. Similar trends are typically described by various analytical models for the charge injection at the metal/semiconductor interface, i.e., thermionic emission (TE),^30^ the modified thermionic emission (MTE) developed for graphene/semiconductor interfaces,^31,32^ direct tunneling, and Fowler–Nordheim (FN) tunneling.^30^ However, in most cases the charge injection at the metal/OSC follows a hybrid process,^33^ which includes tunneling and thermionic emission^34^ with deviations from the standard theory due to defects, surface inhomogeneity, and image-charge barrier lowering, resulting in a bias-dependent barrier height.^30,35^ This hybrid process can be described by a nonideal Schottky diode model with ideality factor *n*,^30^ where the reverse current is *I* = SA**T^2^ exp(−Φ)/*kT*), and where *S* is the contact area, *T* is the absolute temperature, A** is the reduced effective Richardson constant, *k* is the Boltzmann constant, and Φ = Φ_0_ ± (1 – 1/*n*)*qV* is the voltage-dependent energy barrier with nominal barrier height Φ_0_ and ideality factor *n* and where *q* is the elementary charge. The model can be extended to a double barrier system, where different potential barrier heights are formed at two interfaces in series. This model is called the double Schottky barrier (DSB) model and is described by^35^
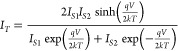
1With reference to the Au/C60/Gr *Vertical Stacks* of this work, *I*_*S*__1,2_ = *S*_1,2_A**T^2^ exp(−Φ_1,2_/*kT*) are the reverse saturation currents, and Φ_1,2_ = Φ_01,02_ ± (1 – 1/*n*_1,2_)*qV* are the voltage-dependent energy barriers at the Gr/C60 and Au/C60 interfaces, respectively. In this model, the current is limited by the reverse currents. When *V* > 0, the Gr/C60 diode (SB_1_) is forward biased and the Au/C60 diode (SB_2_) is reversed biased. Therefore, the measured current is mostly given by the reverse current of SB_2_, i.e., *I*_*S*__2_. On the contrary, when *V* < 0, the current is limited by SB_1_, i.e., *I*_*S*__1_. The DSB model is applied to the *I*–*V*s in Figure 2a considering two different contact surfaces, i.e., *S*_1_ = π(*d*_1_/2)^2^ for the graphene top electrode and *S*_2_ = π(*d*_2_/2)^2^ for the Au bottom electrode, where *d*_1_ and *d*_2_ are the diameter of the contact area and *d*_2_ is 2 μm larger than *d*_1_, while the reduced effective Richardson constant for both interfaces is set to A** = 100 A m^–2^ K^–1^. It is worth observing that A** is extracted from temperature-dependent measurements, and it varies in the range 10^1^–10^3^ A m^–2^ K^–1^ depending on the device and voltage (refer to Figures S6 and S7 and Table S2), as typically reported for metal/OSC interfaces.^33,36^ The variation of the energy barriers extracted with different A** from the aforementioned range is less than 0.1 eV (as shown in Figure S8), and it does not affect the conclusions of this work. Figure 2a shows the DSB fits (red dashed line) of the experimental data. The dashed red line is the average of the DSB fits on five devices per area (the fits of the individual traces are shown in Figure S9). Figure 2b shows the distributions of the energy barriers Φ_01,02_ and ideality factors *n*_1.2_ of the Gr/C60 and Au/C60 interfaces, respectively. The normal distributions are given by Φ_01_ = 0.58 ± 0.01 eV, Φ_02_ = 0.65 ± 0.02 eV, *n*_1_ = 1.033 ± 0.002, and *n*_2_ = 1.036 ± 0.003. The potential barrier height of the Au/C60 interface is similar to previously reported value for C60 field-effect transistors with Au electrodes.^37^ Impedance spectroscopy reveals that the *Vertical Stacks* exhibit the typical nonideal capacitor characteristics. Figure 2c shows the average (on three devices per device area) resistance *R* and capacitance *C* of the *Vertical Stacks* obtained from impedance spectroscopy by fitting the modulus and phase with a nonideal capacitor model, i.e., an *R||C* circuit. Refer to the Supporting Information for details on the fit (Figure S10). The resistance depends on the applied bias, as expected from the *I*–*V* characteristics, while the capacitance is bias-independent, therefore suggesting that the C60 is fully depleted^38^ and that the voltage drops linearly over the whole structure. As a consequence, the electrostatic doping of graphene (i.e., Fermi energy *E*_F_ shift of graphene induced by the applied bias) predicted by the MTE^32^ can be discarded, and the position of the *E*_F_ of the graphene electrode is determined solely by the chemical doping of graphene. It is known that C60 adsorbed on graphene typically results in p-doping.^39^ This is demonstrated by a comparison between field-effect measurements on a set of Gr channels before and after deposition of C60 (Figure S3). The charge neutrality point of the graphene field transistors shifts to positive gate biases (ca. 20 V) when a thin film of C60 is deposited on top of graphene, resulting in an average p-doping of about 1.4 × 10^12^ cm^–2^ (refer to the Supporting Information, eq S1). Figure 2d shows the device resistance and capacitance versus area at a fixed bias of −10 V. The resistance scales as 1/*S*, where *S* is the area of the top electrode, while the capacitance scales as *S* (gray dashed lines in Figure 2d). The static dielectric constant of C60, ϵ_*r*_ = 3.8, is determined from the geometrical capacitance of the device *C* = ϵ_0_ϵ_*r*_*S*/*t*, where *t* is the thickness of the C60 film (80 nm) and ϵ_0_ is the vacuum permittivity. This value is in agreement with previous works.^40,41^ The results of the DSB analysis, supported by the impedance measurements, suggest that the vertical charge transport in the Au/C60/Gr is limited by charge injection. Further insight into the energy barrier and charge injection mechanisms at the Gr/C60 and Au/C60 interfaces is obtained by temperature-dependent measurements.

**Figure 3:**
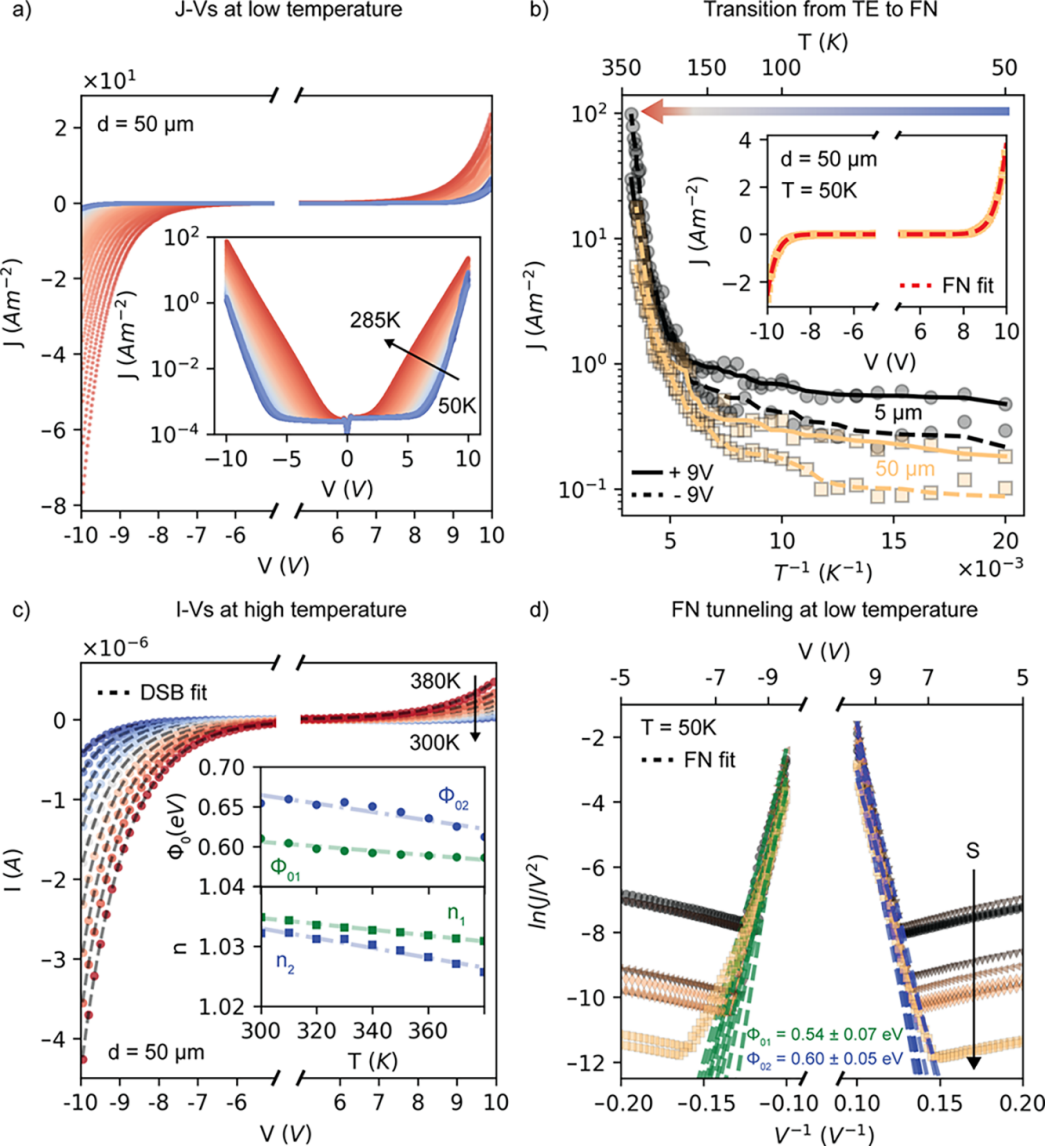
(a) Temperature-dependent *J*–*V* characteristic of a representative 50 μm device from 50 to 285 K. The inset shows the *J*–*V*s on the whole measurement range. (b) Temperature-dependent current density of two representative devices of 5 and 50 μm at fixed biases of ±9 V. The inset shows the FN tunneling model fits for *J*–*V* data at 50 K. (c) Temperature-dependent *I*–*V* characteristic of a representative 50 μm device from 300 to 380 K in steps of 10 K. The inset shows Φ_01_, *n*_1_ and Φ_02_, *n*_2_, extracted from the DSB model, as a function of temperature. (d) ln *J*/*V*^2^ vs 1/*V* plot and FN fits (dashed lines) at 50 K for a set of devices covering the whole diameter range, from 5 to 50 μm. When *V* < 0, charge carriers tunnel through the energy barrier given by the Gr/C60 interface (SB_1_), i.e., Φ_01_. On the contrary, when *V* > 0, charge carriers tunnel through the energy barrier given by the Au/C60 (SB_2_), i.e., Φ_02_.

Temperature-dependent electrical measurements are necessary to distinguish the different charge injection mechanisms contributing to the electrical current in the devices and possibly explain the nonideal thermionic emission observed at room temperature. Low- and high-temperature-dependent electrical measurements are done in two different systems as described in the Methods section, and the results are shown in Figure 3. In particular, Figure 3a shows the *J*–*V* characteristics as a function of temperature of a representative 50 μm device, in linear and logarithmic scale, from 50 to 285 K in steps of 5 K (similar data for a representative 5 μm device are shown in Figure S11); Figure 3c shows the *I*–*V* characteristics for a representative 50 μm device as a function of temperature, from 300 and 380 K in steps of 5 K; Figure 3b shows the current density at ±9 V for two representative devices belonging to the smallest (5 μm) and the largest set (50 μm), from 285 to 50 K; and Figure 3d displays the analysis for the observed low-temperature injection mechanism. Figure 3b shows that while the current density depends strongly on the temperature above 200 K, in agreement with a thermally activated charge injection mechanism, the temperature dependence becomes very weak below 200 K. The current becomes approximately temperature-independent below 100 K. Figure 3c shows the *I*–*V* characteristic of a representative 50 μm in the high-temperature range between 300 and 380 K. In this range, the charge injection is dominated by thermionic emission, and the DSB model can fit all *I*–*V*s. The inset of Figure 3c shows the energy barriers Φ_01_, Φ_02_ and ideality factors *n*_1_, *n*_2_ as a function of temperature, extracted from the DSB fit for the Gr/C60 and Au/C60 interfaces, respectively. The ideality factor of both interfaces decreases with increasing temperature, indicating that the device approaches the ideal TE injection model at higher temperatures, with values around *n*_1_ = 1.026 and *n*_2_ = 1.031. Simultaneously, the potential barrier heights decrease with temperature. This should not come as a surprise, as it is known that barrier inhomogeneity can lead to temperature-dependent energy barriers.^42,43^ Below 100 K, the *J*–*V*s are approximately temperature-independent and well described by the FN tunneling mechanism under the WKB (Wentzel–Kramers–Brillouin) approximation in the formula^30^
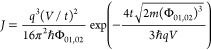
2where *m* is the effective mass of the electron in the semiconductor and ℏ is the reduced Plank constant. Here, the free electron mass is considered. The inset of Figure 3b shows a representative *J*–*V* trace of a representative 50 μm device and the corresponding FN fit, while Figure 3d shows the ln *J*/*V*^2^ vs 1/*V* plot at high bias (typically linear when |*V*| > 8 V) for 5, 10, 15, 20, 25, 30, and 50 μm representative devices at 50 K. The *J*–*V*s are very similar, and the energy barrier of the two interfaces can be derived from the slope of the ln *J*/*V*^2^ vs 1/*V* plot at |*V*| > 8 V, resulting in Φ_01_ = 0.54 ± 0.07 eV and Φ_02_ = 0.60 ± 0.05 eV, which are similar to those obtained from the DSB model fit at high temperature. Refer to the Supporting Information (Figure S12) for the *I*–*V* traces in the range from −10 to 10 V. It is worth observing that the asymmetry of the *J*–*V*s characteristics changes from high to low temperatures. In particular, the rectification ratio at ±9 V, that is *RR* = |(*J*(+9 V)/*J*(−9 V)|, is ca. 1.14 at 50 K and 0.13 at 380 K. Although it is hard to explain quantitatively the phenomenon, one should observe that the injection mechanism changes from nonideal TE at high temperatures to FN tunneling at low temperatures, which are described by different models with different prefactors. The latter are known to deviate significantly from theoretical values in nonideal and/or metal/OSC systems.^33,36^ A transition from FN to direct tunneling would only be possible with a thinner semiconductor. The C60 thickness for transition between FN tunneling and direct tunneling at room temperatures can be approximated by *t* = Φ_B_/*E*, where *E* is the electric field.^30^ Assuming an energy barrier of 0.5 eV, which corresponds roughly to the measured value for the Gr/C60 interface, and an electric field of *E* = 10 V/80 nm = 1.25 MV/cm, this results in a C60 thickness of ∼4 nm. Few-nanometer thin vertical stacks can be theoretically achieved using the architecture and fabrication process described in this work, where graphene is used as the top electrode. In fact, this fabrication process allows to avoid the typical metal atoms intercalation and diffusion observed for metal electrodes evaporated on organic films.^1^ The study of ultrathin stacks could provide insights into the quantum charge transport across OSC/Gr VdW vertical heterostructures, besides potentially enabling FN tunneling at room temperature and therefore making possible the fabrication of organic flexible memories. The latter, which exploit FN tunneling to perform the write and erase operations, could be developed by stacking multiple C60/Gr interfaces.

**Figure 4:**
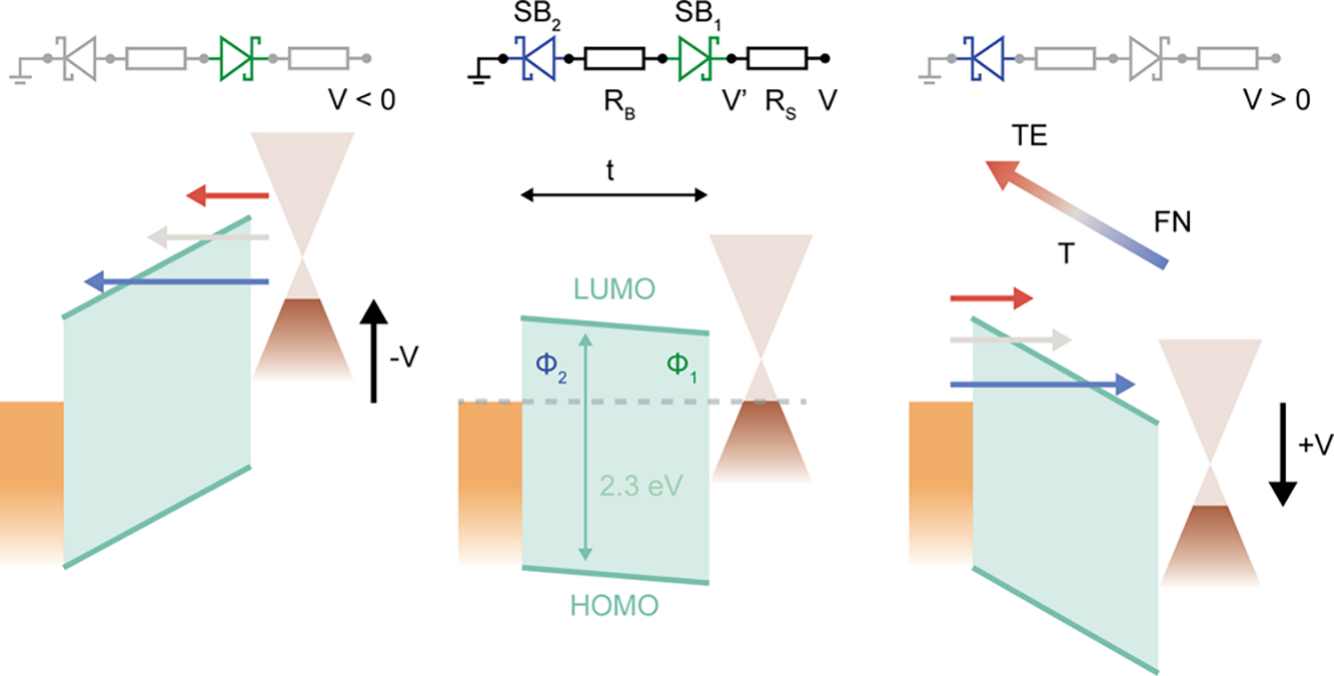
Equivalent electrical circuit model and energy band diagram of the vertical Au/C60/Gr heterostructure. At *V* = 0, the system is at equilibrium, and no current is flowing through the device. When *V* < 0, electrons are injected into the LUMO of the C60 from the graphene electrode. The total current is limited by the reverse current of the Schottky diode SB_1_ of the circuit, which represents the Gr/C60 interface. When *V* > 0, electrons are injected into the LUMO of the C60 from the Au electrode. The total current is limited by the reverse current of the Schottky diode SB_2_, which represents the Au/C60 interface. Contributions of the different injection mechanisms are represented by the colored arrows: FN tunneling is the dominant injection mechanism at low temperature and indicated by the blue arrow, while TE is the dominant injection mechanisms at high temperature and indicated by the red arrow. The gray arrow represents the intermediate regime, where the two contributions are comparable. In the equivalent circuit, *R*_*s*_ is the series resistance due to the graphene electrode and *R*_*B*_ is the bulk resistance of C60. *R*_*s*_ is ca. 20 kΩ at room temperature, as shown by the graphene bridge measurement in the Supporting Information (Figure S2 and Table S1), and therefore the voltage drop is negligible (i.e., *V*′ = *V* – *R*_*s*_*I* ≈ *V*). The C60 is fully depleted, and the voltage drops linearly over the entire structure.

Figure 4 shows the energy band diagram of the vertical Au/C60/Gr heterostructure with the barrier heights extracted from the DSB model at room temperature (above 293 K) and confirmed by the FN model at low temperature (at 50 K). A small built-in potential of roughly 0.07 eV is estimated from the difference between the potential barriers. Assuming that the LUMO and HOMO levels of C60 are located at −4.1 and −6.4 eV, respectively, then (i) the Fermi level of graphene is located at ca. *E*_F,Gr_ = *E*_LUMO_ – Φ_01_ = −4.7 eV, which is possibly shifted with respect to the Dirac point due to p-doping induced by C60 and/or to graphene defects, and (ii) the Au work function is ca. *W* = *E*_LUMO_ – Φ_02_ = −4.8 eV, as typically observed for Au surface with organic adsorbates, due to the pillow effect and/or interface states, which results in an interface dipole and possibly Fermi level pinning at the Au/OSC interface.^44,45^ It is worth observing that because the transport is injection limited, the thermal annealing has an observable effect on the energetics of the barrier: oxygen and moisture desorption affect the surface states, and thus possibly the Fermi level pinning, resulting in more or less conducting devices, as evinced by the *I*–*V* characteristics before and after annealing (Figure S4).

## Conclusions

This work describes the field and thermal induced charge transport mechanisms in Au/C60/Gr VdW vertical heterostructures in the 50–380 K temperature range. Devices having various nominal diameters, which range from 5 to 50 μm, show the same electron transport characteristics. Thus, the fabrication of smaller devices is only limited by the lithography resolution and the fabrication process presented in this work.

Impedance analysis shows that C60 is fully depleted and thus that the voltage drops linearly over the entire structure. The static dielectric constant of C60 extracted from the geometrical capacitance is ϵ_*r*_ = 3.8. The electrical transport measurements across the vertical heterostructure at high temperature (above 300 K) are well described by the DSB model, which gives Φ_01_ = 0.58 ± 0.01 eV, Φ_02_ = 0.65 ± 0.02 eV, *n*_1_ = 1.033 ± 0.002, and *n*_2_ = 1.036 ± 0.003 from the measurements at room temperature (293 K) at the Gr/C60 and Au/C60 interfaces, respectively. At low temperature (50 K), the measurements are well described by FN tunneling, which gives Φ_01_ = 0.54 ± 0.07 eV and Φ_02_ = 0.60 ± 0.05 eV for the Gr/C60 and Au/C60 interfaces. The energy barriers extracted from the two models are very similar. These findings reveal that the charge transport is injection-limited in the temperature range from 50 to 380 K. The current is dominated by TE at high temperature (above 200 K) and limited by FN tunneling at low temperature (below 100 K). The charge transport at intermediate temperature (from room temperature down to 100 K) is possibly described by a hybrid process where thermally excited charge carriers can tunnel through the potential barriers into the LUMO level of C60. From the electrical measurements, direct tunneling is predicted for thin C60 layers of maximum ∼4 nm.

Finally, this study shows that CVD graphene can be used as a top electrode in C60 thin film vertical devices. The fabrication process can potentially be applied to other multilayer hybrid van der Waals heterostructures where the charge transport across neat Gr/OSC interfaces define the functionality of the devices. For instance, tunneling mechanisms could be exploited in high-frequency devices such as organic hot electron transistors, permeable-base transistors, and organic flexible memories.

## Experimental Methods

### Materials

C60 (99.9%) powder was purchased from Sigma-Aldrich and evaporated without further treatments. CVD graphene was grown on copper foils using an in-house automated setup and following the growth protocol which can be found elsewhere.^46−48^

### Fabrication

Devices were fabricated on a Si(525 μm)/SiO_2_ (335 nm) substrate by photolithography and under ambient conditions, as described in the Supporting Information. The chip includes two distinct groups of devices: (i) 119 Au/C60/Gr *Vertical Stacks* (refer to Figure 1a for a schematic of the device structure) and (ii) 34 *Graphene Bridges* (refer to Figure S1). The chip overview is given in Table S3. In both *Vertical Stacks* and *Graphene Bridges*, the C60 thin film is interposed between a bottom gold and a top graphene circular electrodes. In the *Graphene Bridge* architecture, graphene is laterally contacted in order to measure its resistance (see the Supporting Information for the electrical scheme). The chip includes devices having graphene electrodes of various nominal diameter, i.e., 5, 10, 15, 20, 25, 30, and 50 μm, while the Au bottom electrodes are 2 μm larger. In short, Au electrodes were prepatterned and deposited by e-beam physical vapor deposition (EBPVD) and lift-off. Then, the C60 thin film was thermally evaporated (∼0.2 Å/s, 10^–6^ mbar) and patterned by lift-off. Finally, the graphene sheet was wet transferred on the chip and patterned into the top electrode circular shape by reactive ion etching (RIE).

### Electrical Characterization

The electrical measurements were performed under various conditions, i.e., in air, at room temperature in the dark, and under vacuum (∼10^–6^ mbar) in the dark, using the Keithley 4200 semiconductor parameter analyzer. The *I*–*V* characteristics of the *Vertical Stacks* were measured in the −10 to +10 V voltage range, with sweep rate of ca. 1 V/s, steps of 50 mV, and internal averaging of 20 ms. The Au electrode was connected to ground. The graphene resistance was measured in *Graphene Bridge* devices by sweeping the voltage in the −50 to +50 mV voltage range, with sweep rate of ca. 20 mV/s, steps of 1 mV, and internal averaging of 20 ms.

The temperature-dependent *I*–*V*s were measured in the 50–285 K temperature range, in steps of 5 K, using a Lakeshore probe station (CRX-6.5K), under vacuum (∼10^–6^ mbar) and in the dark. The electronics consisted of an AdWin Gold II ADC-DAC and a Femto DDPCA-300 current-to-voltage converter. MATLAB scripts were used for the data acquisition. The *I*–*V* characteristics of the *Vertical Stacks* were measured in the −10 to +10 V voltage range, with sweep rate of ca. 150 mV/s, steps of 10 mV, and with internal averaging of 60 ms. For the charge transport analysis, the backward and forward *I*–*V* sweeps for positive and negative biases were considered, respectively. The full *I*–*V* sweeps (forward and backward) for representative devices measured at room temperature (293 K) and at low temperature (50 K) can be found in Figure S12.

An Agilent 4294a precision impedance analyzer was used to measure the impedance of three representative devices per area. Python scripts were used to control the instrument. The measurement was performed in the dark, under vacuum (∼10^–6^ mbar), and the oscillator level was set to 100 mV in the in the 40 Hz–1 MHz frequency range; 201 data points were acquired. The open/short compensation method was applied to the measurements after the data acquisition and following the Agilent impedance measurement handbook.^49^ Devices for the open/short compensation were embedded on the chip for this scope.

### Raman Spectroscopy

A WITec Alpha 300R confocal Raman microscope was used with a LD 100× objective (Zeiss EC Epiplan-Neofluar Dic, NA = 0.75) and a 300 mm lens-based spectrometer (grating: 600 grooves mm^–1^). Raman spectra were collected with a 532 nm excitation wavelength under ambient conditions. The laser power and integration time of 0.1 mW and 120 s were set for the device, while graphene spectrum on SiO_2_ was acquired using a laser power and integration time of 1 mW and 60 s.

### Atomic Force Microscopy (AFM)

The height image was measured under ambient conditions using a Bruker Icon AFM in tapping mode. The AFM was equipped with a TESPA-V2 cantilever with a tip apex radius of 7 nm, with a resonant frequency 320 kHz and a spring constant of 37 N/m.

### FIB-SEM

The cross section of the representative device was prepared using a FEI Helios 660 G3 UC FIB/SEM system. To protect the graphene and semiconductor layers from damage during the cutting process, a layer of platinum was deposited in two steps: first through electron-induced deposition (3 keV, 800 pA) and then through ion-induced deposition (30 keV, 230 pA). The cross section was then cut in a 30 kV gallium ion beam at an ion current of 47 nA. After cutting, the cross section was polished in sequential steps, decreasing the ion current down to a minimum of 790 pA.

### Modeling, Fitting, and Plotting

Python scripts were developed for the modeling, fitting and visualization of the data. The main libraries that were implemented are (i) scipy curve_fit^50^ for the FN modeling and for the extraction of the graphene resistance, (ii) lmfit^51^ model for the DSB modeling, and (iii) impedance.py^52^ for the impedance spectroscopy analysis.

## Supporting Information Available

Fabrication of Au/C60/Gr heterostructures; FIB/SEM/AFM characterization; electrical transport characterization; chip overview (PDF)

## Abbreviations

OSC: organic semiconductor
Gr: graphene
PMMA: poly(methyl methacrylate)
VdW: van der Waals
C60: buckminsterfullerene
TE: thermionic emission
FE: field emission
FN: Fowler–Nordheim
MTE: modified thermionic emission
DSB: double Schottky barrier
CVD: chemical vapor deposition
WKB: Wentzel–Kramers–Brillouin
OLED: organic light-emitting diode
RF: radio frequency.
