# Supplementary material for: Field and Thermal Emission Limited Charge Injection in Au–C60–Graphene van der Waals Vertical Heterostructures for Organic Electronics
Source: ACS Appl Nano Mater. 2023 May 22;6(11):9444–52. doi: 10.1021/acsanm.3c01090 (PMC10262147; doi:10.1021/acsanm.3c01090)
Supplement: Supplementary file 1 — an3c01090_si_001.pdf [file an3c01090_si_001.pdf]

Supporting information

Field- and Thermal-Emission Limited Charge Injection in Au-C60-Graphene van der Waals Vertical Heterostructures for Organic Electronics

Jacopo Oswald,[a,b] *Davide Beretta,**[a] *Michael Stiefel,*[a] *Roman Furrer,*[a] *Sebastian Lohde,*[a] *Dominique Vuillaume,*[d] *Michel Calame**[a,b,c]

[a] Empa, Swiss Federal Laboratories for Materials Science and Technology, Transport at Nanoscale Interfaces Laboratory, Überlandstrasse 129, CH-8600, Dübendorf, Switzerland

[b] Swiss Nanoscience Institute, University of Basel, Klingelbergstrasse 82, CH-4056, Basel, Switzerland

[c] Department of Physics, University of Basel, Klingelbergstrasse 82, CH-4056, Basel, Switzerland

[d] Institute of Electronic, Microelectronic and Nanotechnology (IEMN), Centre National de la Recherche Scientifique, Villeneuve d'Ascq 59652, France.

* E-mail of the corresponding authors.

Davide Beretta: davide.beretta@empa.ch

Michel Calame: michel.calame@empa.ch

Contents

Fabrication of Au/C60/Gr heterostructures S2
FIB/SEM/AFM characterization S4
Electrical transport characterization S5
Chip overview S19
References S20

Fabrication of Au/C60/Gr heterostructures

The fabrication process is similar to what previously reported by J. Oswald et al. for Au/P3HT/Gr heterostructures[1]. The fabrication consists in the following steps.

a) *Patterning of the bottom electrodes*

Ti (5nm) / Au (30 nm) electrodes are fabricated on a 4 inches Si (525 µm) / SiO_2 (335 nm) wafer, which is pre-cleaned in oxygen plasma (600 W for 5 min). The electrodes (Ti/Au) are deposited by e-beam physical vapour deposition (EBPVD) and patterned by lift-off in DMSO at 100°C for 30 min. The resist for the lift-off (AZ2020nlof) is spin-coated (4000 rpm for 60 s), exposed to UV light (lamp intensity 11 mW/cm^2) through an optical mask, and then developed (AZ726mif, 35 s).

b) *Preparation of the lift-off resist*

The chip with pre-patterned electrodes (Si/SiO_2/Ti/Au) is ultra-sonicated in Acetone for 5 min, rinsed with IPA and blown dry with nitrogen. Then, it is exposed to oxygen plasma at 600 W for 5 min. After HMDS treatment, the chip is coated with a double layer positive optical resist: first, the chip is spin-coated with a LOR5B resist (4000 rpm, 40 s) and backed at 180°C for 5 min. Then, it is spin-coated with an AZ1505 positive resist (4000 rpm, 40 s) and backed at 110°C for 1 min. The device area is exposed for 1.8 s to UV light (lamp intensity 11 mW/cm^2, dose 20 mJ) through an optical mask. Finally, the exposed resist is developed in AZ400K (400K:DIW, 1:4) for 25 s and rinsed with de-ionized water.

c) *Thermal evaporation of C60 and patterning*

A 80 nm film of C60 is obtained by thermal evaporation under vacuum (10^{-6} mbar). The evaporation rate was kept at ~ 0.2 Å/s. The temperature of the substrate was not actively controlled.

d) *CVD graphene transfer*

CVD graphene foil (Cu/Gr/PMMA) is placed to float in a copper etchant (Transene CE-100) for 1h, the PMMA layer facing upwards. Once the copper is completely etched (Gr/PMMA), the etchant is removed and replaced with de-ionized water, twice. Then, the foil is transferred to a 10% HCL cleaning solution for 5 min and transferred back to de-ionized water, twice. The floating graphene foil (Gr/PMMA) is transferred onto the substrate (Si/SiO_2/Au/C60/Gr/PMMA) and let dry in air for 1 h.

e) *PMMA removal from graphene*

The chip is annealed overnight at 80°C in vacuum (~1 mbar). The top PMMA layer is removed in Acetone (5 min) and the chip annealed again overnight at 80°C in vacuum (~1 mbar).

f) *RIE patterning of the graphene top electrode*

The chip is spin-coated with an AZ1505 optical resist (4000 rpm, 40 s) and backed at 110°C for 1 min. The device area is exposed for 1.8 s to UV light (lamp intensity 11 mW/cm2) through an optical mask. The exposed optical resist is developed in AZ400K (400K:DIW, 1:4) for 15 s and rinsed with de-ionized water. Then, RIE is used to remove the unprotected graphene (O_2, 30 sccm, 25 W).

g) *Optical resist removal from graphene*

The optical resist protecting the graphene electrode is removed with Acetone (2 min), then the chip is rinsed in de-ionized water and blown dry with nitrogen.

FIB/SEM/AFM characterization

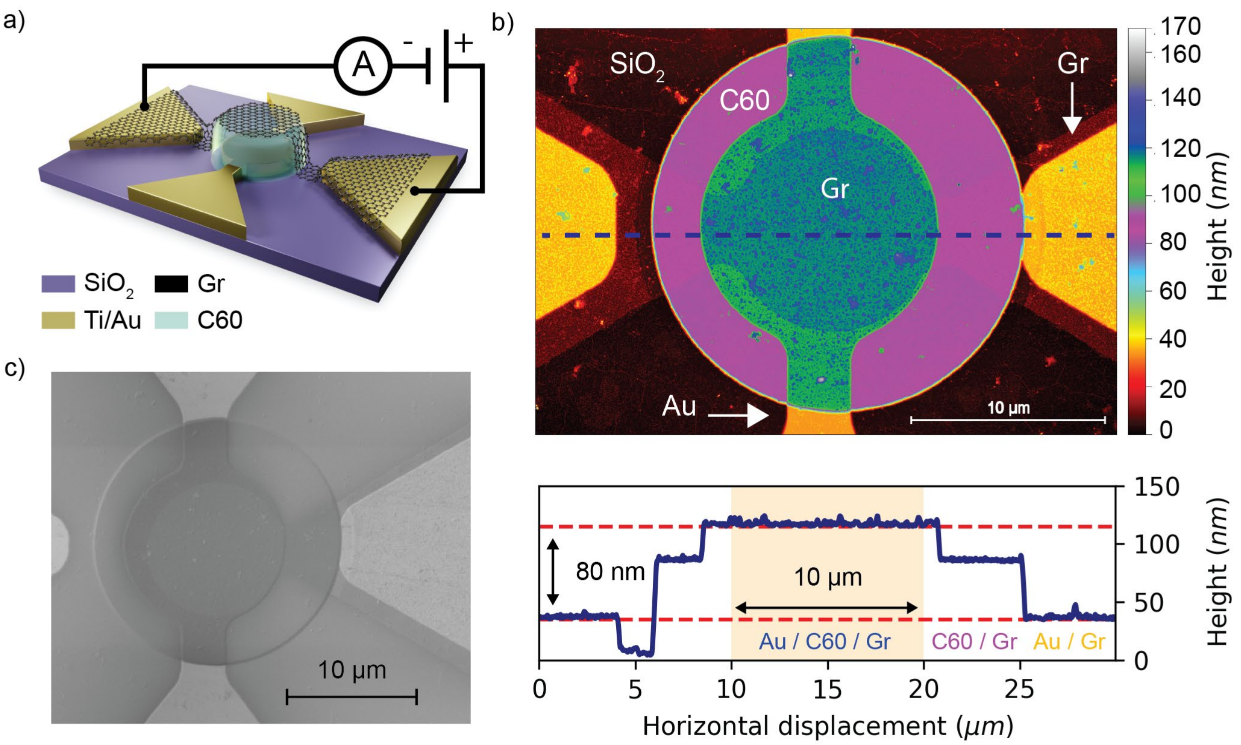

Figure S1. (a) 3D schematic of a representative 10 µm *Graphene Bridge* device (not to scale). Adapted from Oswald, J. et al. ACS Appl. Mater. Interfaces 2022.[1] (b) AFM height image and profile of a representative 10 µm bridge device. (c) SEM image of a representative 10 µm Au/C60/Gr *Vertical Stack* device.

Electrical transport characterization

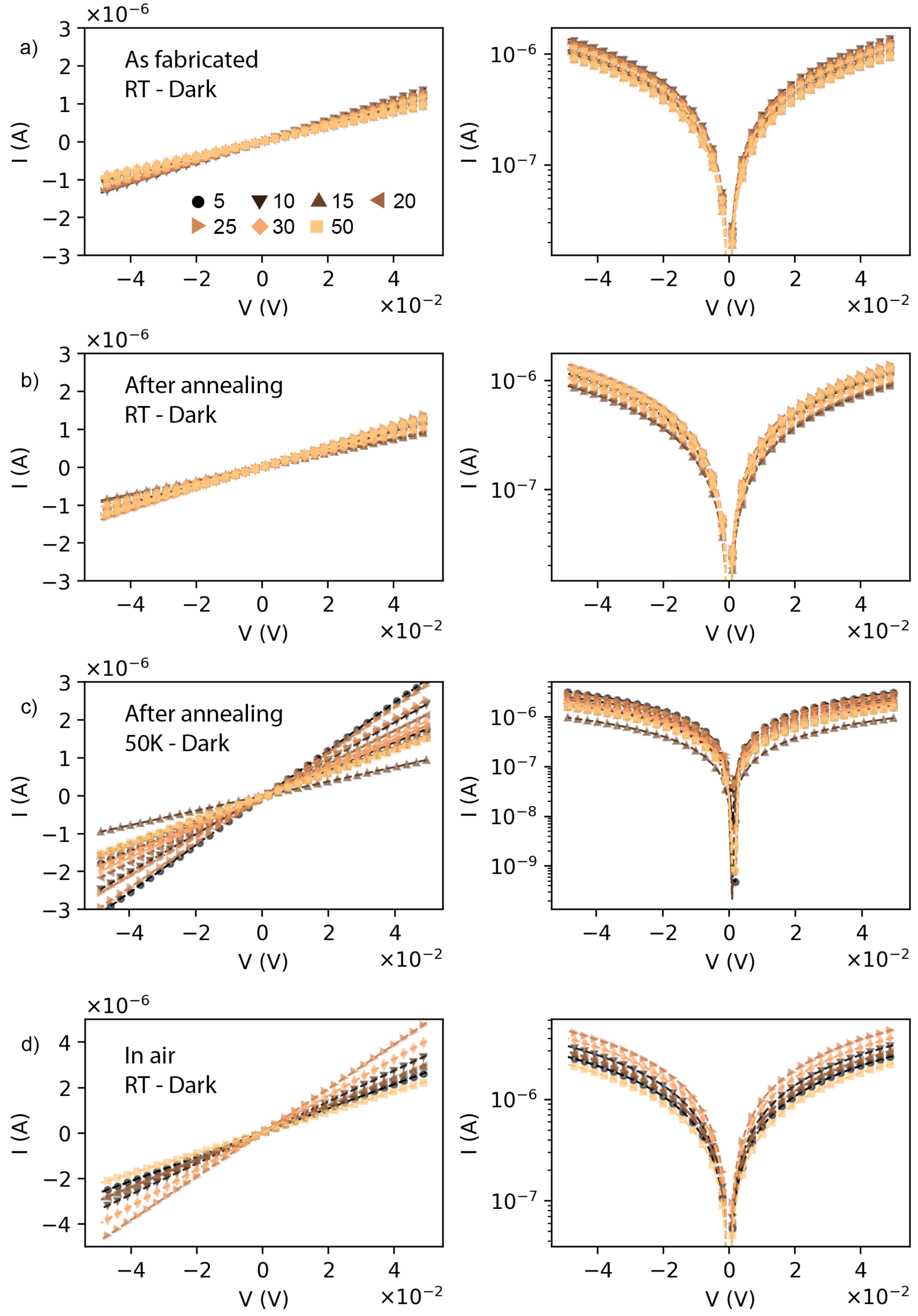

Figure S2. Electrical measurements of the *Graphene Bridges* under different conditions. Left: linear scale. Right: log scale. Symbols represent the diameter of the device. (a) I-V traces of the devices as fabricated measured under vacuum (~10^{-6} mbar), at room temperature (293 K) and in dark. (b) I-V traces of the devices after annealing (110°C for 12h, ~10^{-6} mbar) measured under vacuum (~10^{-6} mbar), at room temperature (293 K), and in dark. (c) I-V traces of the devices after annealing measured under vacuum (~10^{-6} mbar), at 50 K, and in dark. (d) I-V traces of the devices exposed to air. The graphene resistance for a representative device for each diameter, extracted from the linear fit of the I-V traces, is given in Table S1.

	**As fabricated (10^{-6} mbar)**	**After annealing (10^{-6} mbar, 12h 110°C)**		**In Air**
**Device**	RT – 293K, Dark	RT – 293K, Dark	T = 50 K , Dark	RT - 293K, Dark
**5 µm**	42213	42137	27726	18790
**10 µm**	42178	40434	20162	14720
**15 µm**	40343	40536	30875	16718
**20 µm**	41677	41384	22879	16676
**25 µm**	36988	38531	19245	10367
**30 µm**	51914	46965	24877	12350
**50 µm**	52795	46765	31218	22197

Table S1. Graphene resistance (measured in Ω) of a representative *Graphene Bridge* device for each size measured under different environment conditions. Similar resistances are measured for graphene at room temperature (293K) in dark, and after annealing (110°C for 12h) in dark. Lower resistance is found at low temperature (50K), possibly due to higher charge mobility, and in air conditions (293K in dark), as expected from an increased charge carrier density due to the O_2 and H_20 p-doping of graphene. Under all conditions, the resistance of the *Graphene Bridge* is lower than the one of the *Vertical Stack*. Therefore, the series resistance R_s shown in the circuit of Figure 4 can be neglected, and the current across the *Vertical Stack* is limited by the interfaces.

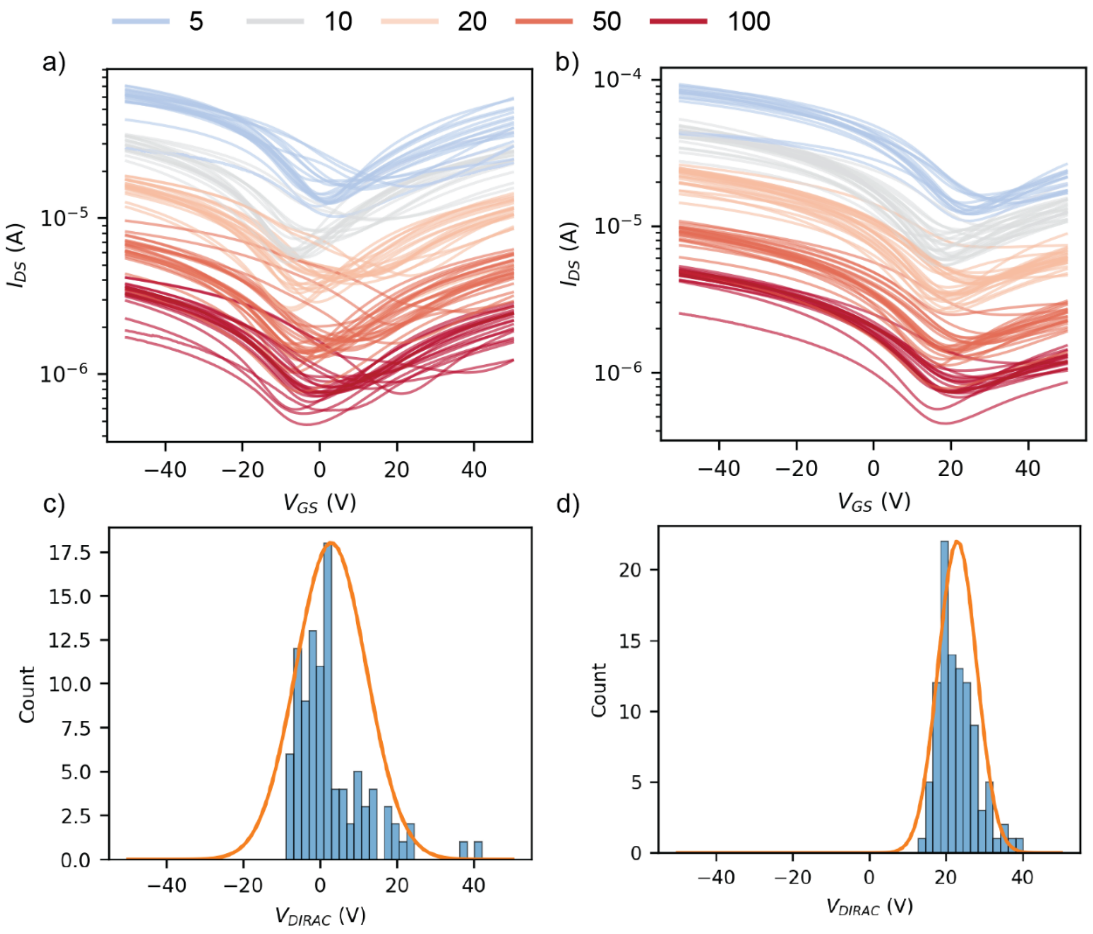

Figure S3. Electrical measurements of graphene field effect transistors under vacuum (~10^{-6} mbar) at room temperature (293K). Channel length is 5 µm while the lengths of the devices are 5, 10, 20, 50 and 100 µm, as shown by the colored lines of (a) and (b). (a) Ids vs. Vgs characteristics of pristine graphene field effect transistors. (b) Ids vs. Vgs characteristics of the graphene field effect transistor doped by C60 (5 nm thin film evaporated on top).

Induced charge carrier density in graphene due to C60

The induced charge carrier density in graphene is deduced from a parallel plate capacitor model, in formula

$$n = C_{gs} \frac{V_{dirac} - V_{gs}}{q} \qquad \text{Eq. S1}$$

Where $C_{gs} = \epsilon_0 \epsilon_r / t$ is the capacitance per unit area, t is the thickness of the oxide (300 nm) and ϵ_r is the dielectric constant of SiO_2 (ca. 3.9). Since the charge neutrality point is shifted by roughly 20 V, as shown in Fig. S3, the induced charge density of graphene doped by C60 is ca. $n \approx 1.4\ x\ 10^{12}\ cm^{-2}$.

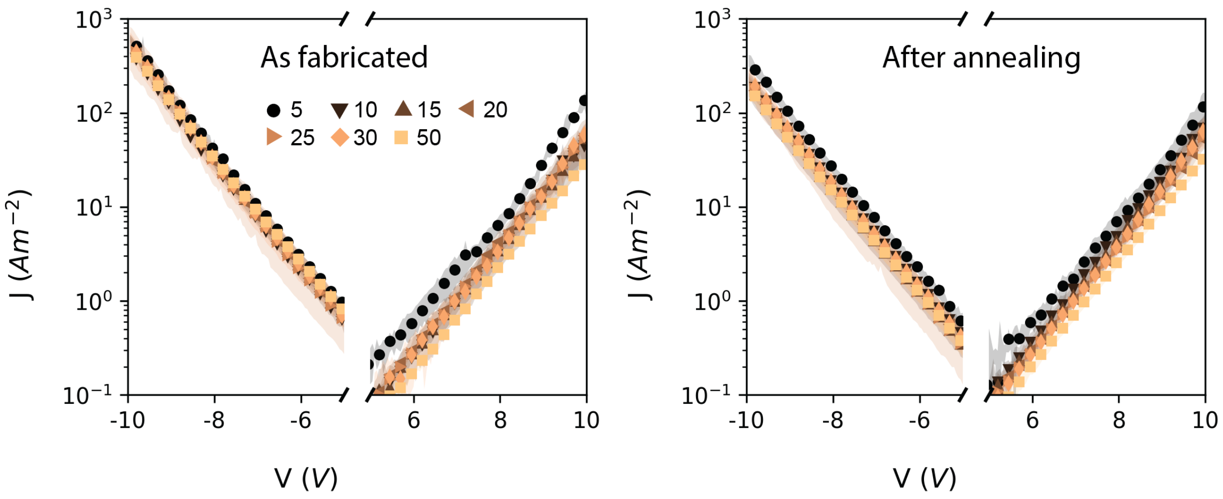

Figure S4. Effect of annealing under vacuum (10^{-6} mbar) on electrical transport across the *Vertical Stack*. (a) J-V traces of the as fabricated devices measured under vacuum (10^{-6} mbar) at room temperature (293 K). (b) J-V traces measured in vacuum at room temperature, after annealing at 110°C for 12h. The graphs show the average of the current-voltage characteristics measured on 2 devices for each diameter: 5, 10, 15, 20, 25, 30, and 50 µm.

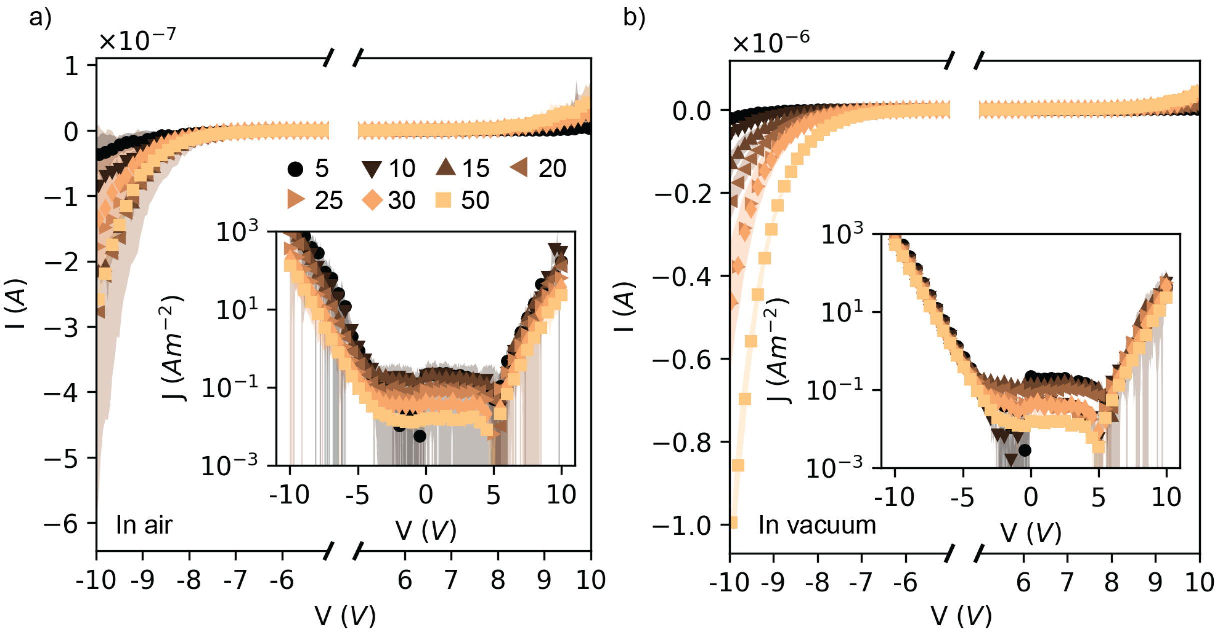

Figure S5. Effect of the environment conditions on the I-V traces of the *Vertical Stack*. (a) Electrical measurements in air at room temperature (293 K). (b) Electrical measurements under vacuum (~10^{-6} mbar) at room temperature (293 K). (a) and (b) are measured on the same set of devices. The graphs show the average of the current-voltage characteristics measured on 2 devices for each diameter: 5, 10, 15, 20, 25, 30, and 50 µm.

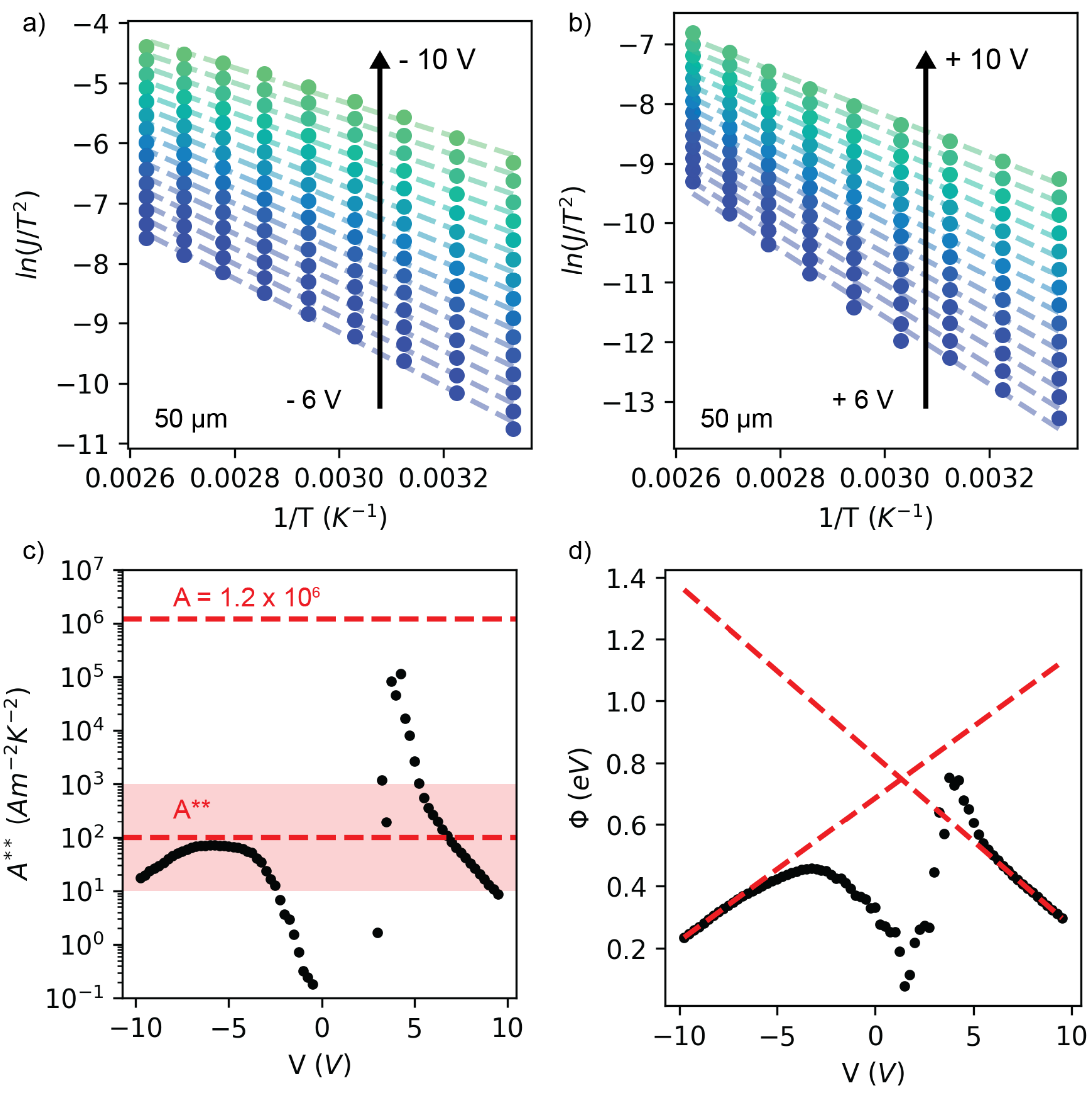

Figure S6. Richardson plot (of the non-ideal Schottky diode model, i.e. $\ln(J/T^2) = \ln(A^{**}_{1,2}) - \Phi_{1,2}(V)/kT$) of a representative 50 µm device for (a) negative applied bias and (b) positive applied bias. For V < 0, the equation represent the reverse current of SB_1 and for V > 0 the equation represent the reverse current of SB_2. In this model the individual interface is considered. (c) Effective Richardson constant $A^{**}_{1,2}$ extracted from the intercept $\ln(A^{**}_{1,2})$ of the Richardson plots, for negative (a) and positive (b) voltages. (d) Potential barrier height as a function of bias $\Phi_{1,2}(V)$, extracted from the slope $-\Phi_{1,2}(V)/k$ of the Richardson plot, for negative (a) and positive (b) voltages. The nominal barrier heights $\Phi_{01,2}$ and ideality factors $n_{1,2}$ are extracted from the intercept and slope of the linear fit of $\Phi_{1,2}(V)$, respectively. Table S2 shows the resulting $\Phi_{01,2}$ and $n_{1,2}$.

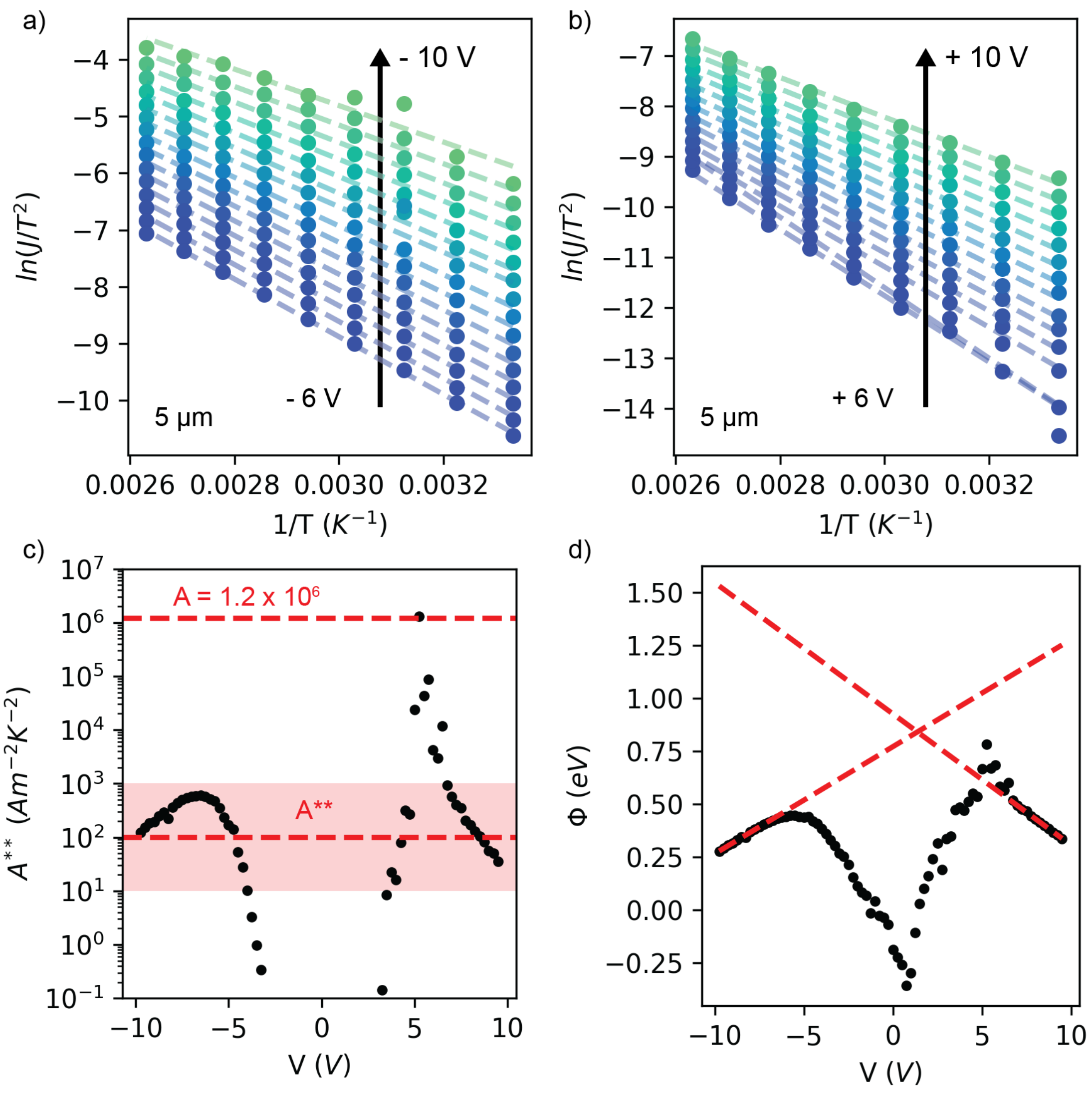

Figure S7. Richardson plot (of the non-ideal Schottky diode model, i.e. $\ln(J/T^2) = \ln(A^{**}_{1,2}) - \Phi_{1,2}(V)/kT$) of a representative 5 μm device for (a) negative applied bias and (b) positive applied bias. For V < 0, the equation represent the reverse current of SB_1 and for V > 0 the equation represent the reverse current of SB_2. In this model the individual interface is considered. (c) Effective Richardson constant $A^{**}_{1,2}$ extracted from the intercept $\ln(A^{**}_{1,2})$ of the Richardson plots, for negative (a) and positive (b) voltages. (d) Potential barrier height as a function of bias $\Phi_{1,2}(V)$, extracted from the slope $-\Phi_{1,2}(V)/k$ of the Richardson plot, for negative (a) and positive (b) voltages. The nominal barrier heights $\Phi_{01,2}$ and ideality factors $n_{1,2}$ are extracted from the intercept and slope of the linear fit of $\Phi_{1,2}(V)$, respectively. Table S2 shows the resulting $\Phi_{01,2}$ and $n_{1,2}$.

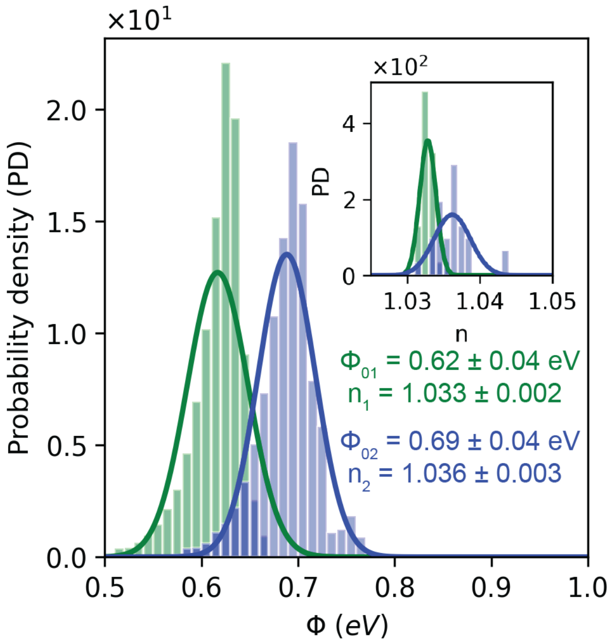

Figure S8. Distribution of $\Phi_{01,02}$ and $n_{1,2}$ extracted from the DSB on 35 device (5 devices for each diameter), associated to the Gr/C60 and Au/C60 interfaces, respectively. Each individual I-V shown in Fig. S9, was fitted using various values of the effective Richardson constant A^{**} varying in the range $10^1 - 10^3\ Am^{-2}K^{-1}$ (in steps of $10\ Am^{-2}K^{-1}$). The extracted energy barrier heights were used to construct the histogram. The variation of the energy barriers extracted with different A^{**} from the aforementioned range is less than 0.1 eV compared to the energy barriers extracted using $A^{**} = 100\ Am^{-2}K^{-1}$ and showed in Fig. 2b.

Discussion on the non-ideal Schottky diode model fitting of individual interfaces (SB_1 and SB_2)

Table S2 shows the ideality factors and energy barriers of SB_1 and SB_2 obtained from the Richardson plots (Fig. S6 and Fig. S7) and considering the non-ideal Schottky diode model with ideality factor n. The resulting effective Richardson constant A^{**} is voltage dependent and vary in the range $10^1 - 10^3\ Am^{-2}K^{-1}$ for |V| > 5 V. The energy barrier and ideality factor of SB_1 are smaller than SB_2 (i.e. $\Phi_{01} < \Phi_2$ and $n_1 < n_2$), following the same trend observed for the results of the DSB model. The higher ideality factors $n_{1,2}$ obtained from the individual Schottky diode model is possibly due to the presence of the second energy barrier and/or to the voltage dependent Richardson constants. This could also lead to a slight overestimation of the energy barriers $\Phi_{01,2}$. For this reason, in the DSB model presented in Figure 2, the Richardson constant was set to the fixed value of $A^{**} = 100\ Am^{-2}K^{-1}$ (red dashed line in Fig. S6 and Fig. S7), and the energy barriers error induced by the Richardson constant, varying in the range $10^1 - 10^3\ Am^{-2}K^{-1}$ (observed range for the individual Schottky diode model, Fig. S6 and Fig. S7), is below 0.1eV, as shown in Figure S8.

**Device**	$\mathbf{n_1}$	$\mathbf{n_2}$	$\mathbf{\Phi_{01}}$ **(eV)**	$\mathbf{\Phi_{02}}$ **(eV)**
**50 um (Fig. S6)**	1.049 ± 0.000	1.058 ± 0.000	0.69 ± 0.00	0.82 ± 0.00
**5 um (Fig. S7)**	1.053 ± 0.002	1.066 ± 0.001	0.77 ± 0.01	0.93 ± 0.01

Table S2. Ideality factors and energy barriers obtained from the Richardson plots of the individual Gr/C60 and Au/C60 interfaces. The data of the 50 μm and 5 μm devices is shown in Fig. 6 and Fig. 7, respectively. The table shows the errors of the linear fit method used to extrapolate the intercept and the slope of the data in Figure S6-7.

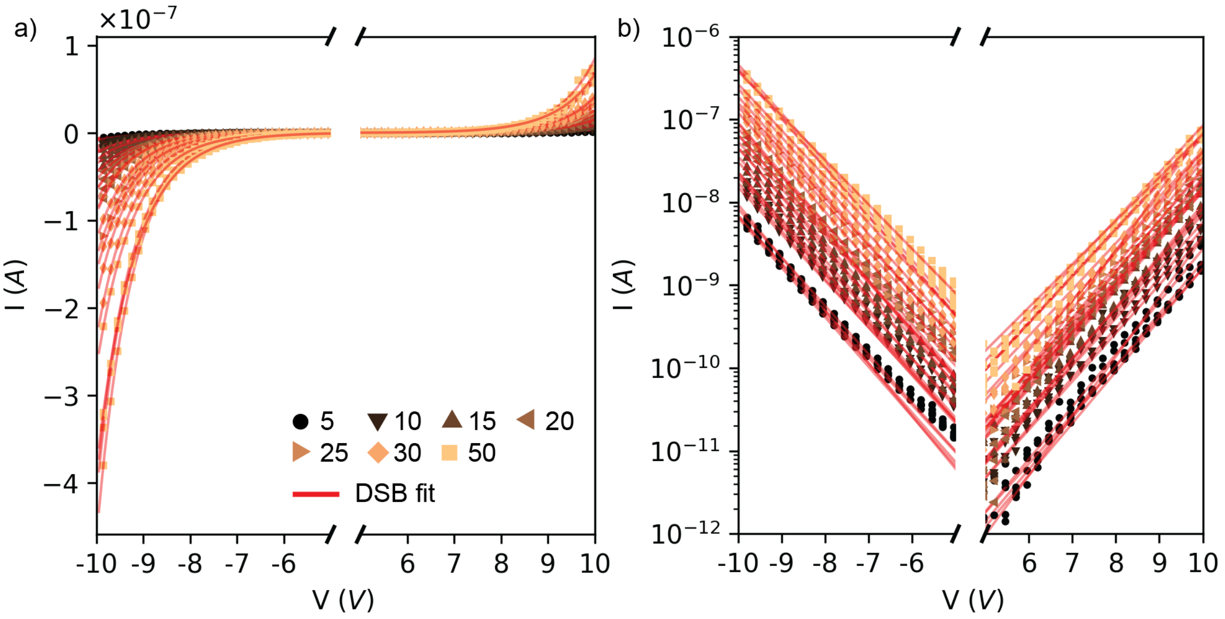

Figure S9. Electrical measurements under vacuum (~10^{-6} mbar) at room temperature (293 K). (a) I-V traces and DSB model fitting (red dashed lines) of the 35 devices (5 devices for each size). (b) Same I-V traces in log scale. The figure does not display the I-V data in the range from -5 V to + 5 V, where the current is below the sensitivity of the instrument (see Figure S12).

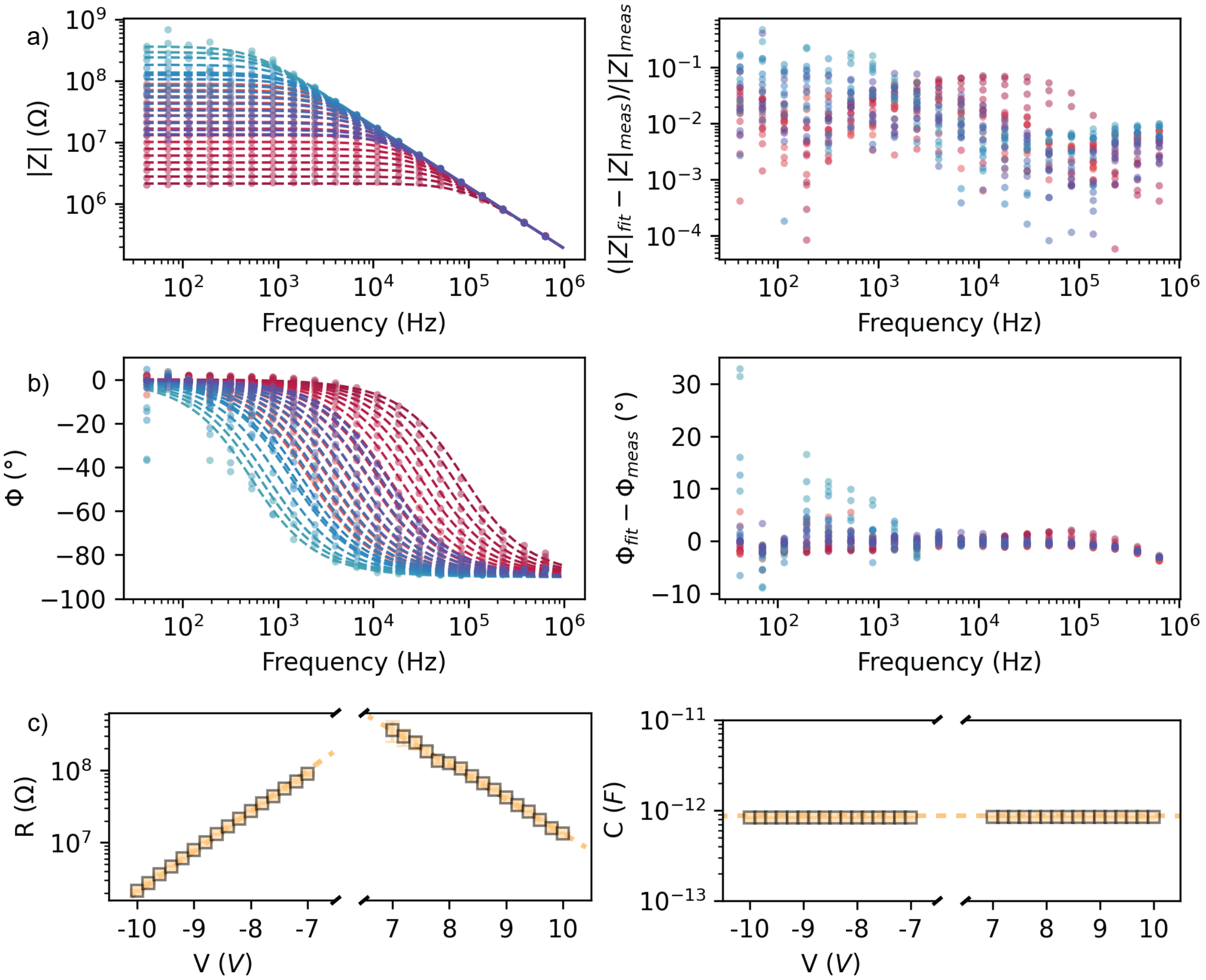

Figure S10. Impedance analysis of a representative 50 µm device. (a) Modulus and R||C model fit (dashed line) of the impedance with relative error. (b) Phase and R||C model fit (dashed line) of the impedance with absolute error. (c) R and C extracted from the fit. The capacitance C is bias independent and therefore, the OSC is fully depleted in the considered voltage range.

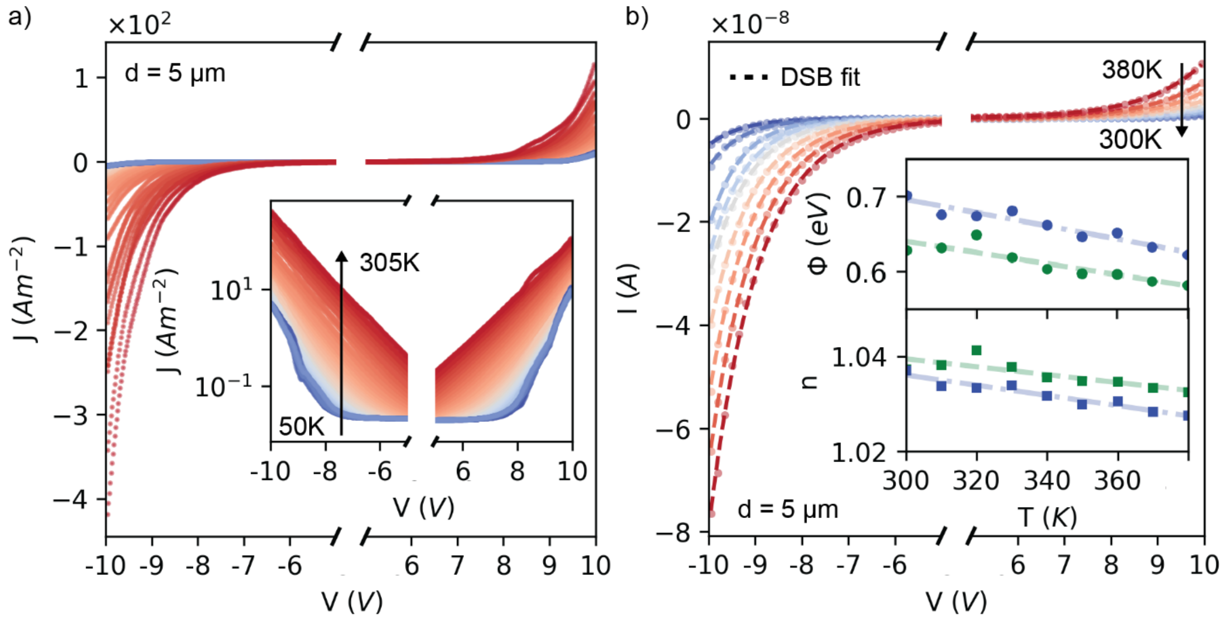

Figure S11. (a) Temperature dependent J-V characteristic of a 5 μm device from 50K to 305K. The inset shows the same traces in log scale. (b) Temperature dependent I-V characteristic of a representative 5 μm device from 300 K to 380 K in steps of 10 K. The inset shows Φ_{01}, n_1 and Φ_{02}, n_2, extracted from the DSB model, as a function of temperature.

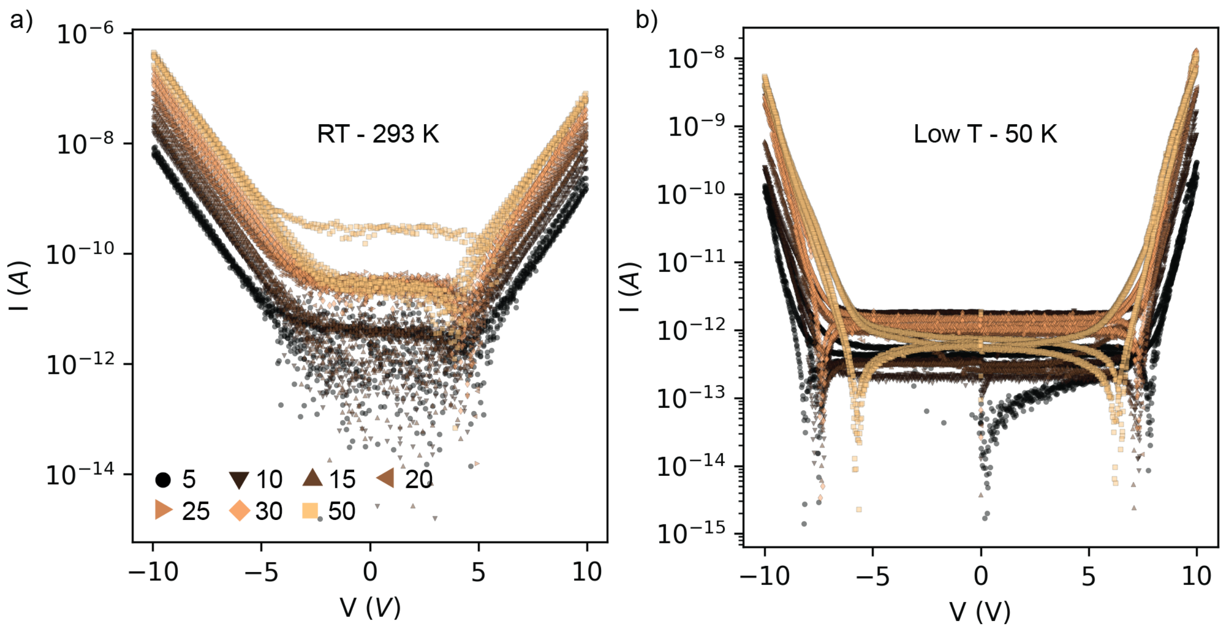

Figure S12. Forward and backward I-V sweeps. (a) I-Vs measured under vacuum at room temperature (293 K) with the Keithley 4200 Semiconductor Parameter Analyzer. The current is below the sensitivity of the instrument in the range from -5 V to + 5 V. (b) I-Vs measured at low temperature (50 K) Lakeshore probe station (CRX-6.5K) operating under vacuum (~10^{-6} mbar). The I-V sweeps show hysteresis in the range from -7 V to + 7V. Forward and backward I-V sweeps are overlapping at higher voltages, where the FN tunneling model is applied.

Chip overview

	A	B	C	D	E	F	G	H	I	J	K	GE diam.	ME diam.
A	AA	AB	AC	AD	AE	AF	AG	AH	AI	AJ	AK	5	7
B	BA	BB	BC	BD	BE	BF	BG	BH	BI	BJ	BK	5	7
C	CA	CB	CC	CD	CE	CF	CG	CH	CI	CJ	CK	5	7
D	DA	DB	DC	DD	DE	DF	DG	DH	DI	DJ	DK	10	12
E	EA	EB	EC	ED	EE	EF	EG	EH	EI	EJ	EK	10	12
F	FA	FB	FC	FD	FE	FF	FG	FH	FI	FJ	FK	15	17
G	GA	GB	GC	GD	GE	GF	GG	GH	GI	GJ	GK	15	17
H	HA	HB	HC	HD	HE	HF	HG	HH	HI	HJ	HK	20	22
I	IA	IB	IC	ID	IE	IF	IG	IH	II	IJ	IK	20	22
J	JA	JB	JC	JD	JE	JF	JG	JH	JI	JJ	JK	20	22
K	KA	KB	KC	KD	KE	KF	KG	KH	KI	KJ	KK	25	27
L	LA	LB	LC	LD	LE	LF	LG	LH	LI	LJ	LK	25	27
M	MA	MB	MC	MD	ME	MF	MG	MH	MI	MJ	MK	30	32
N	NA	NB	NC	ND	NE	NF	NG	NH	NI	NJ	NK	30	32
O	OA	OB	OC	OD	OE	OF	OG	OH	OI	OJ	OK	50	52
P	PA	PB	PC	PD	PE	PF	PG	PH	PI	PJ	PK	50	52
Q	QA	QB	QC	QD	QE	QF	QG	QH	QI	QJ	QK	50	52
Type	Open	Short	Stack	Stack	Stack.	Stack	Stack	Stack	Stack	Bridge	Bridge		

Table S3. Overview of the entire chip. Green cases show the working devices, while the red cases are the not working ones. Roughly, 50% of the chip devices are working and show the same J-V behavior of the device.

References

(1) Oswald, J.; Beretta, D.; Stiefel, M.; Furrer, R.; Romio, A.; Mansour, M. D.; Vuillaume, D.; Calame, M. Charge Transport Across Au–P3HT–Graphene van Der Waals Vertical Heterostructures. *ACS Appl. Mater. Interfaces* **2022**, acsami.2c13148. https://doi.org/10.1021/acsami.2c13148.
